# Genomic mapping of social behavior traits in a F2 cross derived from mice selectively bred for high aggression

**DOI:** 10.1186/1471-2156-11-113

**Published:** 2010-12-31

**Authors:** Derrick L Nehrenberg, Shiliang Wang, Ryan J Buus, James Perkins, Fernando Pardo-Manuel de Villena, Daniel Pomp

**Affiliations:** 1 Department of Genetics, University of North Carolina, Chapel Hill, NC, USA 27599; 2 Department of Cell and Molecular Physiology, University of North Carolina, Chapel Hill, NC, USA 27599; 3 Department of Nutrition, University of North Carolina, Chapel Hill, NC, USA 27599; 4 Carolina Center for Genome Sciences, University of North Carolina, Chapel Hill, NC, USA 27599; 5 Lineberger Comprehensive Cancer Center, University of North Carolina, Chapel Hill, NC, USA 27599

## Abstract

**Background:**

Rapid response to selection was previously observed in mice selected for high levels of inter-male aggression based on number of attacks displayed in a novel social interaction test after isolation housing. Attack levels in this high aggression line (NC900) increased significantly within just four generations of selective breeding, suggesting the presence of a locus with large effect. We conducted an experiment using a small (n ≈ 100) F_2 _cross between the ICR-derived, non-inbred NC900 strain and the low aggression inbred strain C57BL/6J, genotyped for 154 fully informative SNPs, to determine if a locus with large effect controls the high-aggression selection trait. A second goal was to use high density SNP genotyping (n = 549,000) in the parental strains to characterize residual patterns of heterozygosity within NC900, and evaluate regions that are identical by descent (IBD) between NC900 and C57BL/6J, to determine what impacts these may have on accuracy and resolution of quantitative trait locus (QTL) mapping in the F_2 _cross.

**Results:**

No evidence for a locus with major effect on aggressive behavior in mice was identified. However, several QTL with genomewide significance were mapped for aggression on chromosomes 7 and 19 and other social behavior traits on chromosomes 4, 7, 14, and 19. High density genotyping revealed that 28% of the genome is still segregating among the six NC900 females used to originate the F_2 _cross, and that segregating regions are present on every chromosome but are of widely different sizes. Regions of IBD between NC900 and C57BL/6J are found on every chromosome but are most prominent on chromosomes 10, 16 and X. No significant differences were found for amounts of heterozygosity or prevalence of IBD in QTL regions relative to global analysis.

**Conclusions:**

While no major gene was identified to explain the rapid selection response in the NC900 line, transgressive variation (i.e. where the allele from the C57BL/6J increased attack levels) and a significant role for dominant gene action were hallmarks of the genetic architecture for aggressive behavior uncovered in this study. The high levels of heterozygosity and the distribution of minor allele frequency observed in the NC900 population suggest that maintenance of heterozygosity may have been under selection in this line.

## Background

Fighting is a near universal survival trait expressed in animal species as varied as flies [1], mice [2] and humans. Its ubiquity suggests that it serves similar functions. Within a species, fighting may function to disperse its members in ways that reduce pressure on resources necessary for the species to survive [3]. At an individual level, fighting is a strategy for winning competitions for territorial resources necessary for individuals and their relatives to survive [1,2]. For example, the house mouse (*Mus musculus*) regularly patrols the borders of its territory and the highest levels of fighting are observed in areas containing vital resources [4].

A genetic basis for mouse aggression is clearly supported by the success of several different types of selective breeding programs. Divergent selection for attack latency in wild mice [5] and for aggression in Swiss albino [6] and Institute of Cancer Research (ICR; [7]) mice all produced significant differences in aggression levels within 5 generations of selective breeding. Because aggression selection effects occurred so rapidly across several different selective breeding programs, it is reasonable to postulate that the aggression selection response at least partly involves a genetic locus of major effect. Several selection studies have uncovered major genes, including the mini-muscle locus in mice selected for high-levels of voluntary wheel running (HR; [8]) and the high-growth locus [9]. Several mouse aggression QTL have been reported [10,11], and other available evidence to date argues against single locus control of aggression [12,13]. However, it remains possible that QTL with large effects could account for the rapid selection effects of aggression in outbred lines.

The availability of a mouse line (NC900) selectively bred for high levels of attack behavior, created in a high and low aggression selection breeding program from an ICR base population [7], offers a unique opportunity to begin to dissect the genetic architecture of mouse aggression, because in the NC900 lines the number of attacks was the sole selection criterion, whereas in other mouse aggression selection programs attack latencies [5] or rated scores [6] were used. The NC900 selection criterion was attack counts displayed towards a group-reared unselected ICR mouse in a 10-min novel social interaction test following isolation housing at weaning [7]. Levels of aggression in NC900 rapidly diverged from a contemporary low-aggression selection line within just four generations of selective breeding, and these high levels were maintained throughout the long-term selective breeding program [7]. Therefore, we sought to determine whether a locus with large effects controls NC900 male aggression by performing QTL analyses of NC900 social phenotypes.

A straightforward index of attack behavior is to measure its frequency, duration, and latency, and these measures are highly correlated [14]. But in most populations, there will be some mixture of attackers and non-attackers producing both continuous and categorical variables of attack and non-attack. Therefore, we designed a coding system based on the view that there is one continuum of social behavior [15] having appetitive (affiliative in the case of non-agonistic social behaviors) and aversive spectrums. The existence of an aversive, defensive spectrum of behavior is partly supported by evidence that intensity of mouse defensive responses increases in proportion to the intensity of threat [16,17]. However, we recognize that it is not necessarily the case that affiliative and aversive social responses are inversely associated [18] and that agonistic mice are capable of expressing social affiliation at levels comparable to non-agonistic mice [19]. The expression of agonism and non-agonistic sociability are likely a net result complex and perhaps conflicting motivations [20], stemming from somewhat overlapping and distinct biological mechanisms. Thus, assessing indices of affiliation and aversion taken from social interactions may improve our understanding underlying biological differences between purely agonistic, nonagonistic and mixed agonistic/nonagonistic phenotypes.

With this new social interaction coding system as well as the standard measures of attack, our experiments were designed to address questions based on results from a whole genome scan in a small F2 population created by crossing NC900 to the non-aggressive C57BL/6J (B6) inbred line. First, is NC900 aggression controlled by a locus with large effect? Second, can we detect QTL for social behavior measures other than the classic measures of aggression such as frequency and latency of fighting? Finally, we used high-density SNP genotyping in the parental strains to characterize residual patterns of heterozygosity within NC900, and IBD between NC900 and C57BL/6J, to evaluate their potential impacts on QTL mapping in the F_2 _cross. While no major gene was identified to explain the rapid selection response in the NC900 line, transgressive variation and a significant role for dominant gene action were hallmarks of the genetic architecture for aggressive behavior uncovered in this study. We found high levels of residual heterozygosity in the NC900 line, potentially posing limitations in QTL mapping using crosses of non-inbred lines, and suggesting that maintenance of heterozygosity may have been under selection for the high aggression phenotype.

## Results

### Trait statistics

**Figure 1:**
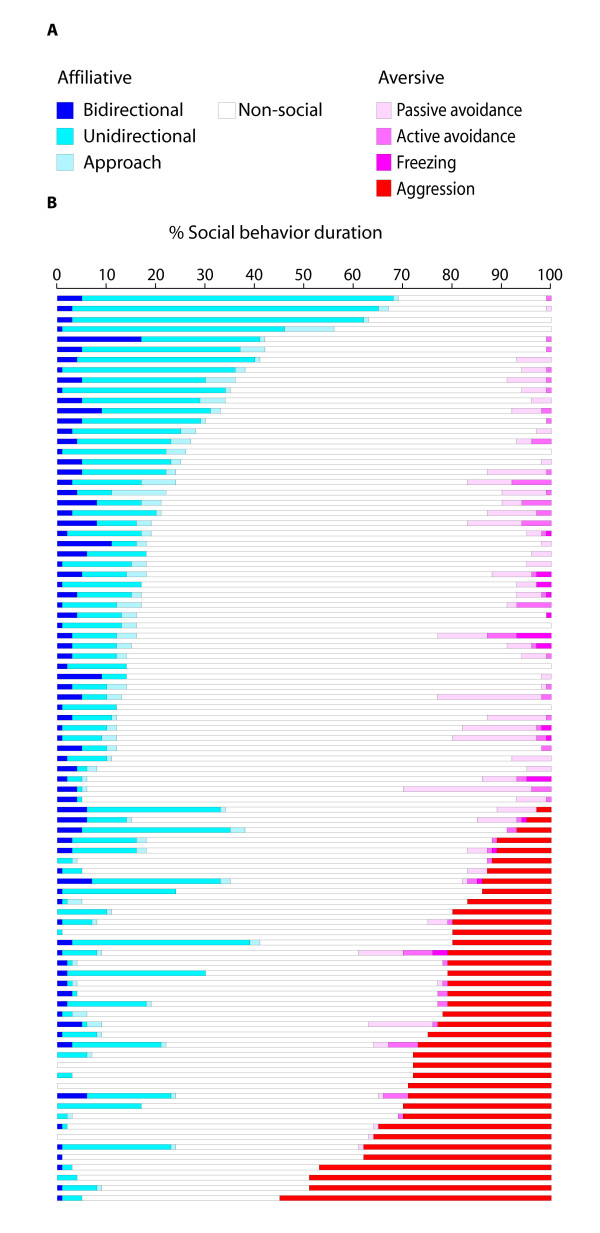
**Behavioral legends and distributions of traits measured in the NC900 × C57BL/6J F2 cross**. **A**. Legend of social behavior traits coded as the percent duration of social test time. **B**. Individual time plots of social behavior percent durations ranked by decreasing percent affiliation levels and increasing percent aggression levels.

**Figure 2:**
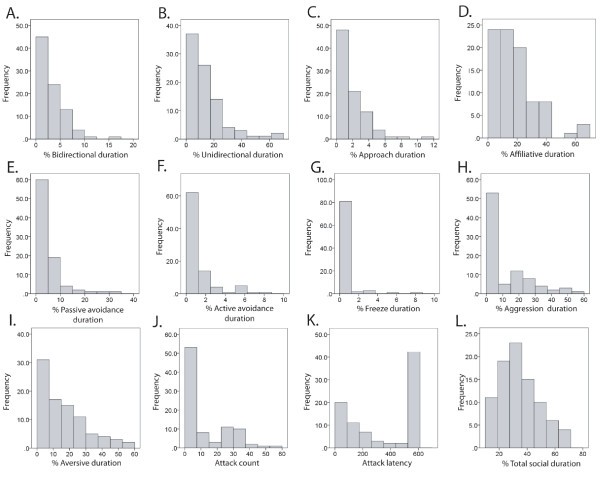
**Histograms illustrating the social behavior trait distributions**. **A**. Percent bidirectional duration, **B**. Percent unidirectional duration, **C**. Percent approach duration, **D**. Percent affiliative duration, **E**. Percent passive avoidance duration, **F**. Percent active avoidance duration, **G**. Percent freezing duration **H**. Percent aggression duration, **I**. Percent aversive duration, **J**. Attack count, **K**. Attack latency, **L**. Percent total social duration

Time plots of percent durations of social behavior phenotypes (Figure 1) illustrate the general relationship between a large range of affiliation and aggression phenotypes captured by the social interaction coding system; as affiliation levels decreased, aggression levels increased. Thirty-eight of the 88 F2 mice (43%) exhibited aggression, and 37 attacked. But, aggressive mice were not devoid of affiliative behavior. Ten of the 37 mice that attacked spent ≥ 23% of the test time exhibiting affiliative behavior, similar to the mean of 23.83% affiliation duration exhibited by nonaggressive mice. Distributions of the social behavior traits are shown in Figure 2 (distributions for behavior counts not shown). All social behavior measures exhibited nonparametric distributions. The prevalence of nonparametric distributions is attributable to the fact that negative counts and durations are not possible, and values close to zero are the mode for most traits. Levels of social behavior expressed by the entire F2 population and subpopulations of aggressive and non-aggressive mice are shown in Table 1. Mean levels of affiliative behaviors differed significantly (*p *< 0.001) between aggressive and nonaggressive mice. Non-aggressive mice spent 76% of their aversive behavior durations displaying passive avoidance, whereas aggressive mice spent 93% of their aversive behavior durations displaying aggression.

**Table 1:** Social behavior phenotype means by non-aggressive and aggressive subpopulations.

Social behavior phenotype	Type	F2	Non-aggression mean (± s.e.)	Aggression mean (± s.e.)
bidirectional	count	4.43 (± 0.39)	5.58 (± 0.48)	2.50 (± 0.49)***
	% duration	3.01 (± 0.31)	3.97 (± 0.42)	1.75 (± 0.33)***
unidirectional	count	10.55 (± 0.74)	12.88 (± 0.91)	6.32 (± 0.96)***
	% duration	13.86 (± 1.44)	17.27 (± 2.10)	9.38 (± 1.65)**
approach	count	5.47 (± 0.53)	7.80 (± 0.73)	2.39 (± 0.41) ***
	% duration	1.83 (± 0.21)	2.59 (± 0.31)	0.84 (± 0.14)***
affiliative	count	19.93 (± 1.37)	26.56 (± 1.51)	11.21 (± 1.64)***
	% duration	18.71 (± 1.59)	23.83 (± 2.14)	11.97 (± 1.91)***
passive avoidance	passive count	3.24 (± 0.42)	4.72 (± 0.59)	1.29 (± 0.23)***
	% duration	3.96 (± 0.64)	5.46 (± 0.81)	1.99 (± 0.94)**
active avoidance	active count	1.97 (± 0.25)	2.24 (± 0.35)	1.61 (± 0.36)
	% duration	1.12 (± 0.19)	1.28 (± 0.27)	0.91 (± 0.26)
freezing	count	0.32 (± 0.09)	0.44 (± 0.15)	0.16 (± 0.09)
	% duration	0.32 (± 0.12)	0.46 (± 0.20)	0.13 (± 0.08)
aggression	count	5.42 (± 0.82)	0.00 (± 0.00)	12.55 (± 1.12)^na^
	% duration	10.73 (± 1.56)	0.00 (± 0.00)	24.85 (± 1.96)^na^
aversive	count	10.94 (± 0.82)	7.40 (± 0.87)	15.61 (± 1.13)***
	% duration	16.13 (± 1.51)	7.19 (± 0.99)	27.88 (± 2.02)***
total social	count	30.87 (± 1.20)	33.96 (1.62)	26.82 (± 1.55)**
	% duration	34.84 (± 1.53)	31.02 (± 1.95)	39.86 (± 2.23)**

attack		17.07 (± 2.83)	0.00 (± 0.00)	39.53 (± 4.41)^na^
attack latency		368.18 (± 26.10)	600.00 (± 0.00)	126.58 (± 18.98)^na^

Superscripts *, ** and *** indicate *t*-test (uncorrected for multiple testing) *p*-values < 0.05, < 0.01 and < than 0.001, respectively. Independent sample t-tests were not performed for aggression counts and aggression duration.

Note - nonaggressive mice are defined as mice that did not perform any aggressive behavior acts defined in Table 7.

Correlations between all the social phenotype traits are shown in Table 2. Attack count was significantly inversely correlated to all types of affiliative social behavior defined by our coding system except unidirectional and approach duration.

**Table 2:** Phenotypic Pearson partial correlations and p-values (with Bonferroni adjustments) for measured traits.

	(2)	(3)	(4)	(5)	(6)	(7)	(8)	(9)	(10)	(11)	(12)
**bidirectional count (1)**	.84***	.45**	.28	.34	.35	.66***	.46**	.22	.15	.13	.17
**% bidirectional duration (2)**		.28	.21	.20	.20	.41*	-.44**	.16	.17	.13	.17
**unidirectional count (3)**			.72***	.63***	.43**	.91***	.76***	.05	-.08	-.03	.08
**% unidirectional duration (4)**				.26	.18	.57	.97***	-.16	-.17	-.14	-.05
**approach count (5)**					.72***	.83***	.37	.34	.14	.22	.25
**% approach duration (6)**						.61***	-.44**	.23	.19	.19	.23
**affiliative count (7)**							.68**	.22	.05	.11	.19
**% affiliative duration (8)**								-.08	-.09	-.07	.01
**passive avoidance count (9)**									.76***	.37	.37
**% passive avoidance duration (10)**										.24	.24
**active avoidance count (11)**											.82***

	**(13)**	**(14)**	**(15)**	**(16)**	**(17)**	**(18)**	**(19)**	**(20)**	**(21)**	**(22)**	
	
**bidirectional count (1)**	.06	.02	-.58***	-.54***	-.42*	-.48***	.47**	.01	-.53***	.42*	
**% bidirectional duration (2)**	-.05	-.02	-.48***	-.44**	-.36	-.36	.29	.07	-.42*	.33	
**unidirectional count (3)**	-.02	-.02	-.55***	.55***	-.54***	-.61***	.68***	.19	-.56***	.52***	
**% unidirectional duration (4)**	-.12	-.11	-.57***	-.34	-.53***	-.44**	.29	.57***	-.37	.26	
**approach count (5)**	.17	.17	-.49***	-.53***	-.23	-.45**	.79***	-.06	-.48**	.61***	
**approach duration (6)**	.12	.12	-.42*	-.44**	-.23	-.34	.54***	.01	-.38	.47**	
**affiliative count (7)**	.07	.06	-.65***	-.66***	-.50***	-.64***	.81***	.08	-.64***	.63***	
**affiliative duration (8)**	-.11	-.09	-.05	-.45*	-.58***	-.51***	.39	-.53***	-.47**	.36	
**passive avoidance count (9)**	.51***	.39	-.45**	-.44**	.24	-.06	.41*	-.14	-.39	.50***	
**passive avoidance duration (10)**	.27	.19	-.35	-.30	.15	.16	.16	.06	-.26	.33	
**active avoidance count (11)**	.23	.27	-.10	-.16	.42*	.06	.41*	-.01	-.13	.17	
**active avoidance duration (12)**	.21	.27	-.15	-.12	.32	.12	.44*	.14	-.16	.13	
**freezing count (13)**		.83***	-.18	-.18	.27	.02	.27	-.09	-.15	.22	
**freezing duration (14)**			-.16	-.15	.21	.04	.21	-.05	-.13	.19	
**aggression count (15)**				.84***	.71***	.69***	-.26	-.31	.76***	-.80***	
**aggression duration (16)**					.54***	.88***	-.39	.40*	.90***	-.83***	
**aversion count (17)**						.68***	.11	.07	.50***	-.47**	
**aversion count (18)**							-.27	.45**	.50***	-.47**	
**total social count (19)**								.14	-.39	.41*	
**% total social duration (20)**									.29	.41*	
**attack (21)**										-.75***	
**attack latency (22)**											

Asterisks *, ** and *** signify correlations exceeding Bonferroni-corrected *p *≥ 0.05, 0.01 and 0.001, respectively.

### F2 social behavior QTL

**Figure 3:**
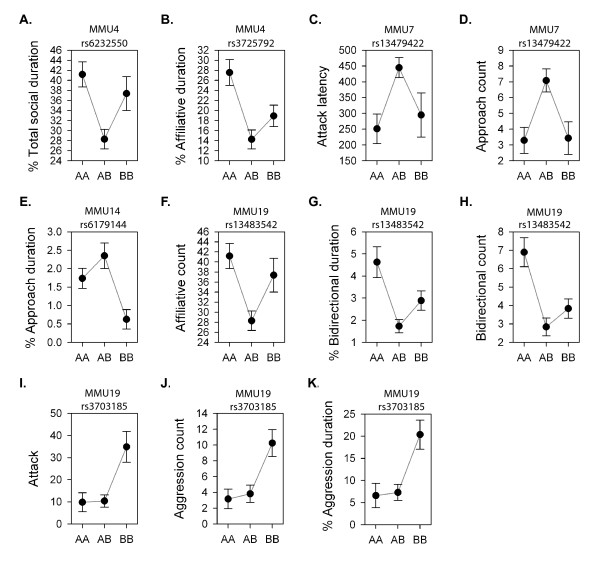
**Plots of social behavior QTL genetic effects where A represents the NC900 allele and B represents the C57BL/6J allele**. **A**. Percent total social duration, **B**. Percent affiliative duration, **C**. Attack latency, **D**. Approach count, **E**. Percent approach duration, **F**. Affiliative count, **G**. Percent bidirectional duration, **H**. Bidirectional count, **I**. Attack, **J**. Aggression count, **K**. Percent aggression duration.

The QTL for social traits detected in this study are presented in Table 3, and their genetic effects are portrayed in Figure 3. Six significant QTL were detected on three different chromosomes, and six suggestive QTL were detected on four different chromosomes. Assuming that a 15-20 cM distance between QTL peaks determines the independence of regions and also assuming pleiotropy for correlated traits, these QTL likely represent four unique loci on four different chromosomes.

**Table 3:** QTL results of genome-wide scans for social interaction QTL in NC900 × B6 F2 mice.

Traits	MMU	PEAK (cM)	LOD	CI	**Additive**^**a**^**(± s.e.)**	**Dominance**^**b**^**(± s.e.)**	% Variation
attack latency	7	49.85	3.10	36.95 - 59.25	-34.09 (± 43.15)	189.30 (± 53.13)	15.91
attack	19	35.46	3.65*	7.76 - 47.36	-12.86 (± 3.51)	-12.10 (± 5.29)	19.21
aggression count	19	35.96	3.41*	20.56 - 48.86	-3.58 (± 1.02)	-2.93 (± 1.56)	17.95

% total social duration	4	26.92	3.12	21.38 - 56.38	2.06 (± 1.90)	-10.00 (± 2.90)	16.49
% affilliative duration	4	30.38	3.32*	37.80 - 56.60	3.81 (± 2.00)	-9.19 (± 3.01)	17.63
approach count	7	51.35	3.22*	40.55 - 59.64	-0.06 (± 0.80)	3.74 (± 1.04)	15.64
% approach duration	14	43.50	2.94	37.80 - 56.60	0.62 (± 0.31)	1.21 (± 0.44)	15.04
affilliative count	19	7.76	3.03	0 - 36.18	4.31 (± 1.57)	-8.97 (± 2.64)	16.00
% bidirectional duration	19	7.76	3.20	0 - 15.26	0.87 (± 0.35)	-2.03 (± 0.59)	17.18
bidirectional count	19	7.76	3.52*	0 - 18.95	0.53 (± 0.43)	-2.52 (± 0.74)	18.84
% aggression duration	19	35.36	3.66*	20.46 - 47.46	-7.15 (± 1.55)	-6.40 (± 2.99)	17.90

Superscript * indicates LOD exceeding 95^th ^percentile LOD permutation threshold. Others exceed the 90^th ^percentile.

^a^Additive effect; a positive value indicates that the increasing allele comes from NC900.

^b^Dominance effect; in relation to the heterozygous genotype and mean of the two homozygous genotype: a positive value indicates that the heterozygote is larger than the midparent (mean of the parents).

A significant QTL for number of attacks was detected on MMU19 with a peak location of 34.5 cM. The attack QTL exhibited both dominance and additive effects, with the B6 allele contributing to an increase in phenotypic value, and underdominance (Figure 3I). Two other QTL, for aggression count and percent aggression duration, also mapped to MMU19 very near the peak for attack (Figure 3J and 3K). Like the QTL for attack, the B6 allele contributed to the increase in the aggression phenotype value, and underdominance was present. A suggestive QTL for attack latency was detected on MMU7 displaying underdominant gene action (Figure 3C).

Two QTL with underdominant gene action were also detected on MMU4: one suggestive QTL for percent total social duration and one significant QTL for percent affiliative duration (Figure 3A and 3B). An overdominant QTL for approach count was detected on MMU7 (Figure 3D) with a QTL peak near the QTL peak for attack latency. A QTL with both additive and dominance effects for percent approach duration was detected on MMU14 (Figure 3E). Three QTL having a peak location at 7.76 cM were detected on MMU19: a suggestive QTL for affiliative count, a suggestive QTL for percent bidirectional duration and a significant QTL for bidirectional count, and all three exhibited underdominant effects (Figure 3F, G, and 3H) with the NC900 allele contributing to the increase in value for the additive component.

In summary, four QTL regions were detected controlling 12 social behavior traits. MMU4 controlled the percent duration of total social behaviors and affiliation. MMU7 controlled both attack latency and approach count. MMU14 controlled percent approach duration. Finally, MMU19 controlled a total of six social behavior traits, including affiliative count, bidirectional count and percent bidirectional duration, attack and aggression count, and percent aggression duration.

### Effects of coat color on social behavior

In the F2 population there were 19 albino, 37 agouti and 32 black mice, which does not significantly differ from the 1:3 ratio expected for the recessive albino trait (χ^2 ^= 0.27, *p *= 0.87). Univariate ANOVA analysis showed that coat color significantly influenced attack latency (*p *< 0.05), and tended to influence approach total (*p *= 0.063) and aggression total (*p *= 0.08). Post hoc analyses with Tukey-adjusted *p*-values showed that albino attack latencies were significantly shorter than those of black mice, and tended to be shorter than those of agouti mice (Table 4).

**Table 4:** Effects of coat color on social behavior means ± s.e in parentheses.

Coat color	attack latency	approach	aggression total
albino	246 (± 55)^a^	3.11 (± 1.00) ^a^	8.74 (± 2.00) ^a^
agouti	395 (± 43)^b^	5.94 (± 0.86) ^a^	5.16 (± 1.44) ^a^
black	407 (± 39)^b^	6.65 (± 6.65) ^a^	3.95 (± 1.02) ^a^

Within columns, means not sharing a common superscript are different (at least *p *< 0.05).

### High-density genotyping

Selection lines are maintained as outbred populations and thus typically segregate for significant but variable regions of the genome. Residual heterozygosity in the NC900 selection line and the amount and distribution of regions of IBD between NC900 and C57BL/6J may have important implications in the interpretation of QTL mapping in our experiment. Therefore, we characterized heterozygosity and haplotype similarity in the F0 parents of our experimental population.

**Figure 4:**
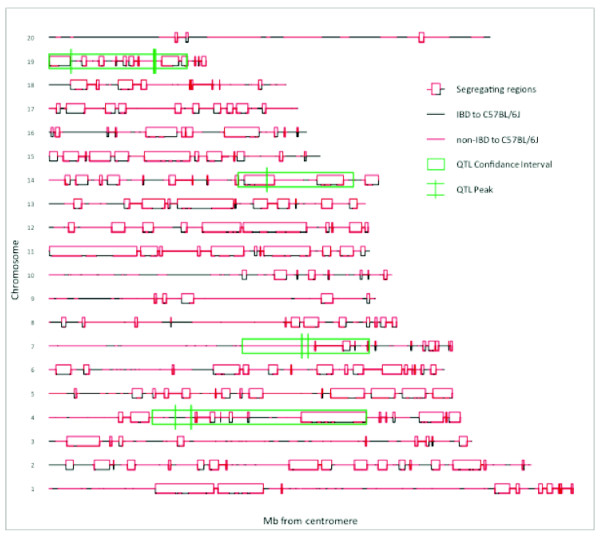
**Segregating regions and identity by descent to C57BL/6J in the six NC900 females used for generation of the F2 males for aggression and social interaction testing**. Segregating regions are shown as open boxes along the chromosome. Regions of IBD to C57BL/6J are denoted by black lines and regions with haplotypes other than C57BL/6J are shown in red. Regions heterozygous for NC900 are shown as open boxes with two red lines are either heterozygous in NC900 breeders or homozygous with two haplotypes that differ from C57BL/6J. Open boxes with a black and red line are either regions heterozygous in NC900 breeders or homozygous with two haplotypes, one different and the other identical to C57BL/6J

**Figure 5:**
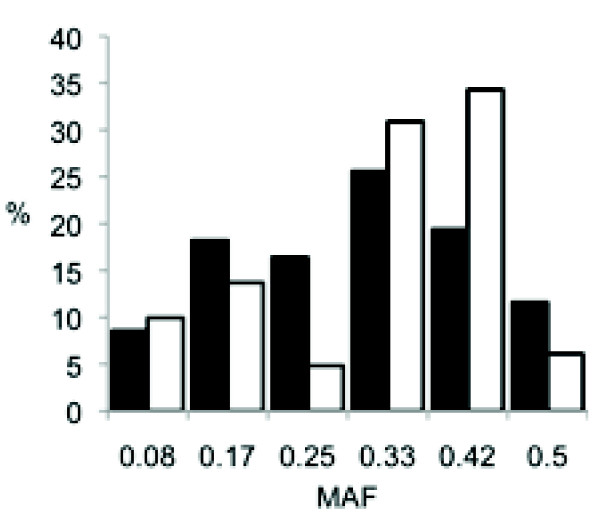
**Frequency and distribution of MAF in segregating regions**. Black bars represent genome-wide analysis and open bars represent the QTL confidence intervals.

Our analysis revealed that 28.3% of the genome is segregating among the six NC900 F0 females. Segregating regions (n = 218) of widely different sizes are present on every chromosome (Figure 4 and Additional File 1, Table S1). However, there are significant differences in the extent of heterozygosity among chromosomes, with chromosomes 11, 15 and 12 harboring the highest levels of heterozygosity (68, 50 and 49%, respectively) while chromosomes X, 7 and 10 have the lowest levels of heterozygosity (2, 6 and 9%, respectively) (Table 5). We have also determined the MAF (minor allele frequency) in every segregating region. MAF follows a bell curve distribution with the mode and mean equal to 0.33 (see black bars on Figure 5). Most segregating regions contain a mix of homozygous and heterozygous NC900 females. Only two segregating regions are lacking heterozygotes among the six NC900 females.

**Table 5:** NC900 female genomewide segregation analysis and C57BL/6J sequence similarity.

	Segregating Regions			Fixed Regions		
**Chr**	**C57BL/6J/** **non-C57BL/6J**	**non-C57BL/6J/** **non-C57BL/6J**	**Total**	**C57BL/6J**	**non-** **C57BL/6J**	**Total**

1	9.47 (4.88)	41.2 (21.2)	50.7 (26.1)	23.1 (11.9)	120 (61.9)	143 (73.8)
2	14.3 (8.01)	43.7 (24.5)	58.1 (32.5)	27.7 (15.5)	92.8 (51.9)	120 (67.4)
3	3.19 (2.04)	19.5 (12.4)	22.7 (14.5)	39.5 (25.2)	94.3 (60.2)	133 (85.4)
4	19.0 (12.4)	32.6 (21.4)	51.7 (33.8)	37.2 (24.4)	63.5 (41.6)	100 (66.1)
5	15.4 (10.3)	41.4 (27.7)	56.9 (38.0)	29.5 (19.7)	63.0 (42.1)	92.6 (61.9)
6	18.5 (12.6)	27.6 (18.8)	46.2 (31.5)	27.3 (18.6)	72.9 (49.7)	100 (68.4)
7	4.14 (2.76)	5.35 (3.57)	9.49 (6.34)	33.2 (22.2)	106 (71.4)	140 (93.6)
8	6.47 (5.03)	13.9 (10.8)	20.4 (15.8)	32.3 (25.1)	75.9 (59.0)	108 (84.1)
9	5.03 (4.16)	6.57 (5.43)	11.6 (9.59)	24.4 (20.2)	84.9 (70.2)	109 (90.4)
10	3.99 (3.14)	7.21 (5.67)	11.2 (8.82)	54.0 (42.5)	61.6 (48.5)	115 (91.1)
11	25.5 (21.5)	54.9 (46.2)	80.5 (67.7)	6.05 (5.09)	32.2 (27.1)	38.2 (32.2)
12	15.3 (12.9)	43.0 (36.4)	58.3 (49.3)	10.1 (8.56)	49.7 (42.0)	59.8 (50.6)
13	26.3 (22.4)	25.2 (21.5)	51.6 (44)	21.8 (18.5)	43.8 (37.3)	65.6 (55.9)
14	17.7 (14.5)	24.3 (19.9)	42.1 (34.5)	26.4 (21.6)	53.5 (43.8)	79.9 (65.4)
15	20.0 (19.9)	30.3 (30.1)	50.3 (50.1)	15.9 (15.8)	34.1 (33.9)	50.1 (49.8)
16	10.9 (11.4)	22.5 (23.6)	33.4 (35.1)	38.9 (40.8)	22.8 (24.0)	61.8 (64.8)
17	16.8 (18.3)	12.4 (13.5)	29.3 (31.8)	13.9 (15.1)	48.7 (52.9)	62.7 (68.1)
18	5.47 (6.24)	10.9 (12.5)	16.4 (18.7)	17.6 (20.1)	53.6 (61.1)	71.2 (81.2)
19	10.3 (17.6)	14.6 (25.1)	24.9 (42.8)	10.0 (17.1)	23.2 (39.9)	33.2 (57.1)
X	0.77 (0.47)	2.85 (1.74)	3.62 (2.21)	88.7 (54.3)	70.9 (43.4)	159 (97.7)

Total	249 (9.66)	480 (18.6)	730 (28.3)	578 (22.4)	1269 (49.2)	1848 (71.6)

Note - Numbers represent Mb while numbers in parenthesis are percentages.

We found no evidence of regions with more than two haplotypes segregating in the NC900 population. Therefore, it is simple to identify regions of IBD between NC900 and C57BL/6J independently of the heterozygosity status. Regions of IBD are found on every chromosome but are most prominent on chromosomes X, 10, and 16 (54, 43 and 41%, respectively) (Table 5).

We have combined the heterozygosity and IBD analyses to generate a map of the expected inheritance patterns in our experimental F2 population. At one extreme are homozygous regions in the NC900 females that are also IBD to C57BL/6J. In these regions (22% of the genome) polymorphisms should be limited to de novo mutations accumulated since the divergence of the two strains. On the other extreme are regions of heterozygosity in the NC900 females with two haplotypes that differ from C57BL/6J. In these regions, the mapping population can have as many as six different genotypes and their frequency depends on the MAF in the six NC900 females (Figure 5 and Additional File 2, Figure S1).

We characterized the heterozygosity, IBD, and MAF in the QTL confidence intervals identified. We did not find significant differences for amounts of heterozygosity or prevalence of IBD when compared to global analysis (compare Tables 5 and 6). Conversely, the MAF are shifted toward higher values in QTL confidence intervals (Figure 5).

**Table 6:** Local analysis in the QTL confidence intervals.

	Segregating Regions			Fixed Regions		
**Chr**	**C57BL/6J/** **non-C57BL/6J**	**non-C57BL/6J/** **non-C57BL/6J**	**Total**	**C57BL/6J**	**non-C57BL/6J**	**Total**

4	15.3 (19.3)	13.0 (16.5)	28.4 (35.8)	29.3 (36.9)	21.5 (27.1)	50.8 (64.1)
7	2.50 (5.31)	0.68 (1.44)	3.18 (6.76)	4.10 (8.73)	39.7 (84.5)	43.8 (93.2)
14	5.98 (13.9)	15.2 (35.6)	21.2 (49.5)	4.97 (11.6)	16.5 (38.7)	21.5 (50.4)
19	10.1 (19.9)	12.5 (24.5)	22.6 (44.4)	9.32 (18.2)	19.0 (37.2)	28.3 (55.5)

Total	33.9 (15.4)	41.5 (18.8)	75.4 (34.2)	47.7 (21.6)	96.9 (44.0)	144. (65.7)

Note - Numbers represent Mb, while numbers in parenthesis are percentages.

## Discussion

Our first objective was to test whether the aggression phenotype of NC900 mice is controlled primarily by a locus with large effect using a small NC900 × B6 F2 cross well powered to detect QTL with large effect. While no such result was identified, we were able to map QTL for social behavior traits despite a relative lack of power to detect loci with modest effect. A single QTL for aggression (percent duration count and attack count) was detected on MMU19 but had a paradoxical genetic effect. The B6 parental line, which rarely exhibits aggression in our social interaction test, contributed the high aggression allele. While QTL describing transgressive variation (i.e. in this case where the allele from the C57BL/6J, rather than the NC900 increased attack levels) are relatively common (Rieseberg, 1999), they are usually detected within a broad architecture dominated by QTL allelic effects that are in the expected direction based on parental phenotypic divergence [21-25]. Our results suggest that the NC900 mouse inter-male aggression phenotype is likely controlled by many additional QTL, each having effects too small to detect in the present F2 population.

Consideration of NC900 aggression as a complex trait is consistent with the genetic architecture of aggression in Drosophila where a minimum of 5 aggression QTL and extensive epistasis were detected [13]. A cross of NC900 and NC100, while technically more difficult to evaluate (e.g. allele sharing), would be required to rule out the possibility that a genetic locus with a large effect was segregating in the ICR base population and contributed to the NC900 selection response, but is fixed in the same direction in the B6 line and thus would not be detected in this cross. The aggression QTL we detected do not correspond to those previously reported in mice [10,11], which is not surprising for several reasons: 1) different methods of aggression behavior measurement; 2) different pre-aggression test housing environments; 3) laboratory-to-laboratory variation, and 4) different genetic background. It is particularly important to note that the aggression QTL we detected are potentially unique to the type of open field, social novelty aggression testing we used vs. resident intruder test [10]. As an example, Roubertoux and colleagues found that differences in rearing and testing conditions produced stark differences in the genetic correlates of aggressive behavior [11].

Extensive dominance gene action was evident for aggression traits, and significant over/under-dominance effects for other social behavior traits were also evident. Strong heterosis may imply that these traits are relevant to overall fitness [26]. From an evolutionary perspective, social behaviors like intermale aggression are likely important components of fitness, because they affect the ability to produce offspring. In a laboratory setting, dominant males sire > 90% of the offspring [27], but it is not known to what degree social behavior like aggression impacts fitness in wild mice. We have observed that overdominant gene action is common for other traits that affect overall fitness in mice such as litter size [22].

We observed qualitative differences in individual attack styles. Highly aggressive mice were noted for higher attack speed and more attacks described as front attacks. We did not attempt to assess individual differences in counts of front, side, and rear attacks in this study, because these types of attacks were realized retrospectively. We also lacked means to measure attack speed in the present study. Given the degree of the striking qualitative differences in attack speed and style we observed, such measures are warranted in the future. Future studies should also consider the possibility that the genotypes of the standard opponents indirectly interact with the genotypes of the subjects [28]. We selected B6 standard opponents to in an effort to avoid confounding aggressive subject vs. aggressive social partner interactions, but did not account for F2 phenotypic variation that may be attributable to indirect genetic interactions between B6 standard opponent genotypes and various mixtures of NC900 × B6 F2 genotypes.

The peaks of QTL for attack latency and approach on MMU7 fall ~10 Mb from the *tyrosinase *gene (*tyr*). This is noteworthy because it has long been known that the *tyr *locus is associated with behavioral differences [29]. Since NC900 mice are albino, and albinism is caused by a recessive point mutation in *tyr *[30], resulting in the absence of melanin production in the hair, skin, and eyes, it is conceivable that coat color represents a marker for attack latency and approach. Even though attack latency was never a NC900 high-aggression selection criterion, mean attack latencies significantly decreased across generations of selection for high levels of attack (unpublished data). We have reported effects of coat color on wheel running speed and also detected a QTL controlling mouse voluntary wheel running speed linked to *tyr *locus [31]. In addition, effects of coat color on open-field activity have been reported [32,33] and a contextual fear conditioning QTL has also been associated with the *tyr *locus [34]. It is tempting to speculate that attack latency, wheel running speed, and fear conditioning QTL linked to the *tyr *locus share common functional variation manifested by control over the pace with which animals perform motivated behaviors like attack, fear conditioning, and wheel running. Alternatively, the attack latency QTL could relate to visual defects caused by the absence of melanin. For example, it is feasible that a lack of melanin could augment sensitivity to light in albino mice [35] resulting in an enhanced light-induced stress response. Thus, the faster attack latencies observed by F2 albino mice may be attributable to indirect effects of the *tyr *locus on the stress response rather than direct effects of *tyr *attack latency and approach tendencies per se.

High-density genotyping can be used to identify regions that are identical by descent (IBD) between strains used in mapping populations to refine QTL candidate intervals. Regions of IBD within QTL confidence intervals should be excluded as candidate QTL regions while regions with more than one haplotype remain. Based on our analysis, we can exclude 22% of the QTL confidence intervals indentified in this study.

QTL mapping was performed using methods that assume that our mapping population is analogous to a standard F2 population derived from two inbred strains. In contrast with these assumptions, our high-density genotype analysis shows that one-third of the genome is, in fact, segregating in a more complex pattern. This complex pattern poses challenges in QTL mapping especially in crosses with widely variable MAF distributions across the genome (Additional File 2, Figure S1). We suggest that crosses involving selection lines use a combination of markers that distinguish genetic variation both between and within the selection lines. Ignoring segregating regions is likely to result in overlooking loci that have been otherwise identified. This should be particularly relevant to populations in which heterozygosity has been selected for a particular phenotype. The size of the segregating regions, the presence of a very narrow bottleneck of only 3 breeding pairs (see Materials and Methods), and the distribution of MAF in the six NC900 females used in this study suggest that maintenance of heterozygosity may have been under selection during creation of the high-aggression NC900 line. Furthermore, the size of the segregating regions is smaller than in other selection lines derived from similar ICR stock and genotyped with the same high-density platform (FPMV and DP unpublished).

## Conclusions

If a locus with large effect does not control aggression, how can the genetic basis of NC900 aggression be determined? It is important to recognize that the only NC900 selection criterion is number of attacks displayed in a novel social interaction test after isolation housing. Group housed NC900 animals display significantly less aggression than isolated NC900 mice [36]. This gene by environment interaction is not unique to NC900 mice. Aggression phenotypes are strongly influenced by many environmental factors including group dynamics [37], maternal social relationships [38] and childhood maltreatment [39]. Considering that predisposition to violence can require genotype (e.g. low-activity *MAOA*) and environmental (severe maltreatment) interactions [40], we should expect that aggression phenotypes pivot on complex relationships between genetic, environmental, and ontogenetic sources of variance [41]. The challenge is to unravel the complexity. Given the reliability with which an environmental factor (i.e. isolation housing) can induce aggression in mice [42], it is an ideal phenotypic target for dissecting how a complex behavioral trait develops. But if aggression is controlled by many QTL that each have a small effect and whose influence is only apparent in particular environmental conditions, and is characterized by extensive epitasis [13], then very large populations of segregating genotypes along with contrasting environmental conditions will be needed to understand its genetic architecture.

The size of the segregating regions, the presence of a very narrow bottleneck of only 3 breeding pairs (see Materials and Methods), and the distribution of MAF in the six NC900 females used in this study suggest that maintenance of heterozygosity may have been under selection during the creation of the high-aggression NC900 lines. While lack of complete pedigrees and the limited sample size in this study prevents us from reaching definitive conclusions, this speculative hypothesis bears further study as such a result would have important ramifications for the nature of selection response for behavioral phenotypes.

## Methods

### Mouse Lines

The NC900 mouse line was selectively bred for high-aggression using only one selection criterion - attack counts displayed by an isolation housed 45 day old male toward a group-housed partner mouse in a 10-min novel social interaction test [7]. At generation 55 (in 2004), 3 families of NC900 mice were successfully rederived to produce specific pathogen-free mice. Selective breeding for social interaction attack count did not proceed for every subsequent generation due to space and logistical constraints, but retention of phenotype was confirmed by periodically assessing isolated male NC900 attack counts against a group-housed C57BL/6J (B6; Jackson Laboratory, Bar Harbor, ME) mouse. At the suggestion of the UNC-Chapel Hill Institutional Animal Care and Use Committee, the age for standard social interaction testing was increased from 45 d to a minimum of 8 weeks. B6 mice were selected as social partners and as F2 founders because they rarely attack or display any observable form of aggression in our social interaction test (data not shown).

All mice were housed in standard cages on a 10:14 hr light/dark cycle (18:00 - 08:00 dark) and provided *ad libitum *access to feed and water. Mice were fed Prolab Isopro RMH 3000 (Lab Diet: protein 26%, fat 14%, carbohydrates 60%) through the experimental period. All procedures were conducted in accordance with NIH guidelines for the care and use of experimental animals and based on protocols approved by the Institutional Animal Care and Use Committee of UNC-Chapel Hill.

### F2 cross

Six NC900 females having brothers that previously tested positive for high aggression (≥ 20 attacks within a 10 minute social interaction test against a B6 social partner) were mated with six B6 males in single mating pairs. Fifty-nine F1 animals were produced (28 females, 31 males). At 21 days of age all F1 males were weaned into isolation housing and tested at 8-9 weeks of age in the social interaction test against 8 to 10 week old B6 males. Eighteen of the 31 F1 males attacked at least once, and all 6 families produced attacking males. The 18 attacking males, plus five other males that did not attack within 5 minutes, but did attack within 10 minutes (as per the NC900 aggression selection criteria), were bred to randomly selected, non-sibling F1 females in 23 single mating groups. Twenty F1 mating groups produced 100 F2 males, which were weaned into isolation housing at 21 days of age.

### Social behavior phenotyping

At 8-9 weeks of age, F1 and F2 males were tested against group-housed 8-10 week old B6 mice in the social interaction test according to the standard aggression selection procedures (Cairns et al., 1983), except for the social test duration. Social interaction tests were conducted in a 20 × 21 × 31 cm Plexiglas open field arena containing bed-o-cob bedding and lit to 360 Lux during 1900 - 2300 hours of the dark cycle. During the initial 2 minutes, the partner and subject animals habituated to opposing sides of the field separated by an opaque sliding divider. Upon removing the divider, mice interacted freely for 5 minutes. Black F2 subject mice and black B6 partner mice were differentiated by marking B6 partners with black permanent marker at the tips of their tails. Tests were recorded using a digital video camera (Sony DCR-SR45). Digital video files were coded using Noldus Observer XT™ software (Leesburg, VA). Subject coat color and subject and social partner body weights were recorded immediately after the social interaction test.

Attack behavior was defined as a vigorous lunge accompanied by a bite on the social partner animal [7]. Aggressive behavior included: bite, chase, lunge, tail-rattling, and feints. Attack behavior was coded as a point variable within states of aggression. The social interaction coding system was developed in three stages. First, we performed *ad-hoc *scoring of NC900 and B6 social interactions behaviors in an effort to identify all social behaviors exhibited by the parental strains. Next, we developed a pilot coding system that could capture the range of behaviors observed in the ad-hoc observations. This pilot coding system was then further refined and tested by two coders using a subset of the F2 social interaction tests in an iterative manner until a high-degree of inter-coder reliability was established for all behavior codes (Cohen's κ > 0.80; [43]) using reliability statistic analyses included in Observer XT. The entire F2 population was coded by a single individual who was periodically tested for maintenance of intra-coder reliability against coded video standards (Cohen's κ > 0.90) using Observer XT throughout the F2 social interaction-coding period.

The final set of social interaction measures are defined in Table 7. This system is designed to measure behavioral states as subcategories of affiliative and aversive responses. Four behavioral subcategories comprised aversive responses: passive avoidance, active avoidance, freezing and aggression (Figure 1A). We chose to measure these behaviors because they are readily identifiable and naturally occurring defensive behaviors in mice [16,17]. Affiliative behavior codes were more difficult to create because mouse affiliation is largely comprised of nonagonistic physical contact intermingled with bouts of sniffing and grooming. Therefore, our rationale for creating the social affiliation measures was simply to reliably capture categories of non-agonistic behavior. We found we could reliably extract three categories. Subjects could move toward partners (approach), direct nonagonistic contact towards the partner (unidirectional), or be in a state of mutual nonagonistic contact with the partner (bidirectional). It is important to note that affiliative social behaviors were only coded when the subject was actively engaging the partner (and vice-versa in the case of bidirectional behavior); passive affiliative contact was not coded. Thus, three behavioral subcategories comprised affiliative behavior: approach, unidirectional, and bidirectional (Figure 1A). Subcategories of affiliative and aversive behaviors were coded as mutually exclusive behavioral states. Social tests were typically coded in one pass at real-time speed. Social interaction bouts that occurred too quickly to detect in real-time were coded at slower speeds.

**Table 7:** Definition of social interaction phenotypes

Calculated Phenotype	Phenotype	Definition
^a ^Affiliative - sum of bidirectional, unidirectional, and approach behavior	^a ^bidirectional	simultaneous display of affiliative behaviors by the subject and partner mouse (e.g. simultaneous facial sniffing, grooming, anogenital sniffing), irrespective of which mouse initiated the contact
	^a ^unidirectional	affiliative behavior displayed by subject with no observable response from partner
	^a ^approach	subject walks towards or follows partner (not coded if attack behavior immediately follows)

	^a ^nonsocial	no subject-partner interaction

^a ^Aversive - sum of passive avoidance, active avoidance, freezing, and aggression	^a ^passive avoidance	partner-initiated affiliative social contact passively ignored by subject without moving away
	^a ^active avoidance	partner-initiated affiliative social contact actively ignored by subject by moving away
	^a ^freezing	crouched, prone, & immobile posture lasting ≥ 1.0 second
	^a ^aggression	vigorous lunge and bite typically directed at the partners' flanks and back, but also including lunges, feints, chasing, tail-rattling, and bites without lunge.

	attack	vigorous lunge and bite
	attack latency	amount of time expired from beginning of the social interaction test till the first attack

^a ^Percent duration and counts coded for each variable.

### Low-density Genotyping and Linkage Map

A total of 100 F2 mice and eight F0 parental mice (six NC900 dams and two B6 sires) were genotyped for 176 SNPs using matrix-assisted laser desorption ionization-time of flight mass spectrometry (MALDI-TOF MS). SNPs were initially selected based on their relatively even spacing across the genome and their predicted complete informativeness between NC900 and B6 mice, using data from the Wellcome-CTC Mouse Strain SNP Genotype Set http://www.well.ox.ac.uk/mouse/INBREDS. Predicted informativeness was based on genotypes from 8 mice representing lines (M16, ICR; [25]) derived from the same general genetic background as the ICR population used as the base for selection of the NC900 lines, relative to the genotype of B6. After genotyping, we excluded SNPs showing allele sharing across NC900 and B6 parents, and SNPs whose F2 genotypic frequencies significantly departed from the χ^2 ^distribution based on Mendelian expectation. The final set of 154 SNPs used for QTL analyses is provided in Additional File 3, Table S2.

### QTL analyses

Eighty-eight of the 100 F2 subjects produced were analyzed; six were excluded because B6 partners displayed aggression towards the subject and six additional F2 subjects were excluded due to incomplete genotype data. Histograms of social behavior were plotted to determine normality of traits using SPSS 16.0 (Chicago, IL). Social behavior trait means between aggressive and non-aggressive subpopulations of the F2 mice were compared by t-tests, and the effects of coat color on social behavior traits were assessed by ANOVA with Tukey-adjusted *p*-values using SPSS 16.0. Social behavior Pearson partial correlations with Bonferonni corrections were generated with SAS 9.1 (Cary, NC).

Genome-wide QTL scans and stepwise model selections were performed using the R/qtl package in the R 2.7.2 environment [44]. We used the stepwise model selection procedure to determine the influences of fixed effects (experimental batch), random effects (dam) and covariates (litter size and number of brothers and sisters, subject weight and partner body weight). Since none of the social behaviors were significantly influenced by any of these effects or covariates, they were not included in the single QTL model genome-wide scans. All LOD significance thresholds were determined by permutation [45], and LOD scores exceeding the permuted 95^th ^and 90^th ^percentiles were deemed significant and suggestive, respectively. Additive and dominance effects were extracted using R/qtl.

The social behavior measures exhibited non-parametric distributions. Therefore we performed non-parametric QTL scans by specifying the non-parametric distribution of traits which is equivalent to Kruskal-Wallis test statistic [46]. The ranks of the phenotypic values, rather than the phenotypic values themselves, were fitted into a standard linear regression model to extract the percent variation and *p*-values for the QTL.

### High-density Genotyping

High quality, high-molecular weight DNA was extracted from the six NC900 females used for generation of the F2 population used in this study using phenol-chlororform. Samples were normalized to 50 ng/ul, processed according to the Affymetrix Genome-Wide Human SNP Nsp/Sty Assay Kit 5.0/6.0 protocol and hybridized to the Affymetrix Mouse Diversity genotyping array at the Functional Genomics core at the University of North Carolina at Chapel Hill. A total of 549,000 SNPS were used in this study.

SNP genotype calling was performed as described previously [47]. To identify regions segregating within the founders of the experimental population, we determined the frequency of H calls with 200 consecutive SNP windows in each one of the six NC900 females independently. Regions with > 2% of SNPs with heterozygous calls were deemed heterozygous based on the analysis of 101 fully and partially inbred strains from the Jackson Laboratory (FPMV unpublished). We used a similar approach to identify regions of homozygosity in the six NC900 females containing different haplotypes (i.e., regions segregating among NC900 females). We mapped the start and end positions of each segregating interval to the first heterozygous SNP for segregating regions within a mouse and to the first SNP with discordant genotypes in segregating regions among the six NC900 females. This approach provides a conservative estimate of the length and position of heterozygous regions. For each segregating region we also determined the MAF.

IBD between each of the six NC900 females and C57BL/6J was determined by analysis of SNP markers. Analysis was performed using a 100 SNP marker sliding window and a threshold of 98% identity to C57BL/6J. This threshold is based on the characterization of multiple sister strains and biological duplicates (FPMV unpublished). Boundaries of the regions of IBD were determined. QTL regions were converted from CM to bp using the Center for Genome Dynamics Mouse Map Converter [48].

## Authors' contributions

DLN contributed to development and maintenance of the NC900 line, created and phenotyped the F2 cross, and drafted most of the manuscript. SW conducted the QTL statistical analyses. RJB conducted the high-density genotyping and drafted related sections of the manuscript. JP assisted with phenotypic data collection. FPMdV participated in design of the study and provided interpretation of the high-density genotyping results. DP conceived of the study, participated in its design and coordination, and helped to draft the manuscript. All authors read and approved the final manuscript.

## Supplementary Material

Additional file 1 **Table S1**. Size and chromosome distribution of segregating regions among NC900 breeders. Click here for file

Additional file 2 **Figure S1**. Regional MAF analysis. Segregating regions were analyzed for MAF. Regions are classified with a MAF of 1 - 6 and are displayed as plateaus corresponding to the segregating region. Click here for file

Additional file 3 **Table S2**. List of 154 SNPs used in the final NC900 × B6 F2 map with known physical (Mb) and linkage (cM) positions from the Wellcome-CTC Mouse Strain SNP Genotype Set http://www.well.ox.ac.uk/mouse/INBREDS. Click here for file
