# Supplementary material for: Genomic mapping of social behavior traits in a F2 cross derived from mice selectively bred for high aggression
Source: BMC Genet. 2010 Dec 31;11:113. doi: 10.1186/1471-2156-11-113 (PMC3022667; doi:10.1186/1471-2156-11-113)
Supplement: Additional file 1 — Table S1. Size and chromosome distribution of segregating regions among NC900 breeders. [file 1471-2156-11-113-S1.DOC]

**Additional File, Table S1.** Size and chromosome distribution of segregating regions among NC900 breeders.

| **Chr** | **Average size (Mb)** | **Total size (Mb)** | **Regions Segregating** |
| --- | --- | --- | --- |
| 1 | 5.07 | 50.7 | 10 |
| 2 | 3.22 | 58.1 | 18 |
| 3 | 2.52 | 22.7 | 9 |
| 4 | 3.69 | 51.7 | 14 |
| 5 | 4.06 | 56.9 | 14 |
| 6 | 2.88 | 46.2 | 16 |
| 7 | 0.86 | 9.49 | 11 |
| 8 | 1.7 | 20.4 | 12 |
| 9 | 2.32 | 11.6 | 5 |
| 10 | 1.4 | 11.2 | 8 |
| 11 | 7.32 | 80.5 | 11 |
| 12 | 5.83 | 58.3 | 10 |
| 13 | 4.69 | 51.6 | 11 |
| 14 | 3.51 | 42.1 | 12 |
| 15 | 4.19 | 50.3 | 12 |
| 16 | 3.04 | 33.4 | 11 |
| 17 | 2.93 | 29.3 | 10 |
| 18 | 1.82 | 16.4 | 9 |
| 19 | 2.08 | 24.9 | 12 |
| 20 | 1.2 | 3.62 | 3 |
| Total | 3.34 | 730 | 218 |
