# Supplementary figures and images for: Genomic mapping of social behavior traits in a F2 cross derived from mice selectively bred for high aggression
Source: BMC Genet. 2010 Dec 31;11:113. doi: 10.1186/1471-2156-11-113 (PMC3022667; doi:10.1186/1471-2156-11-113)

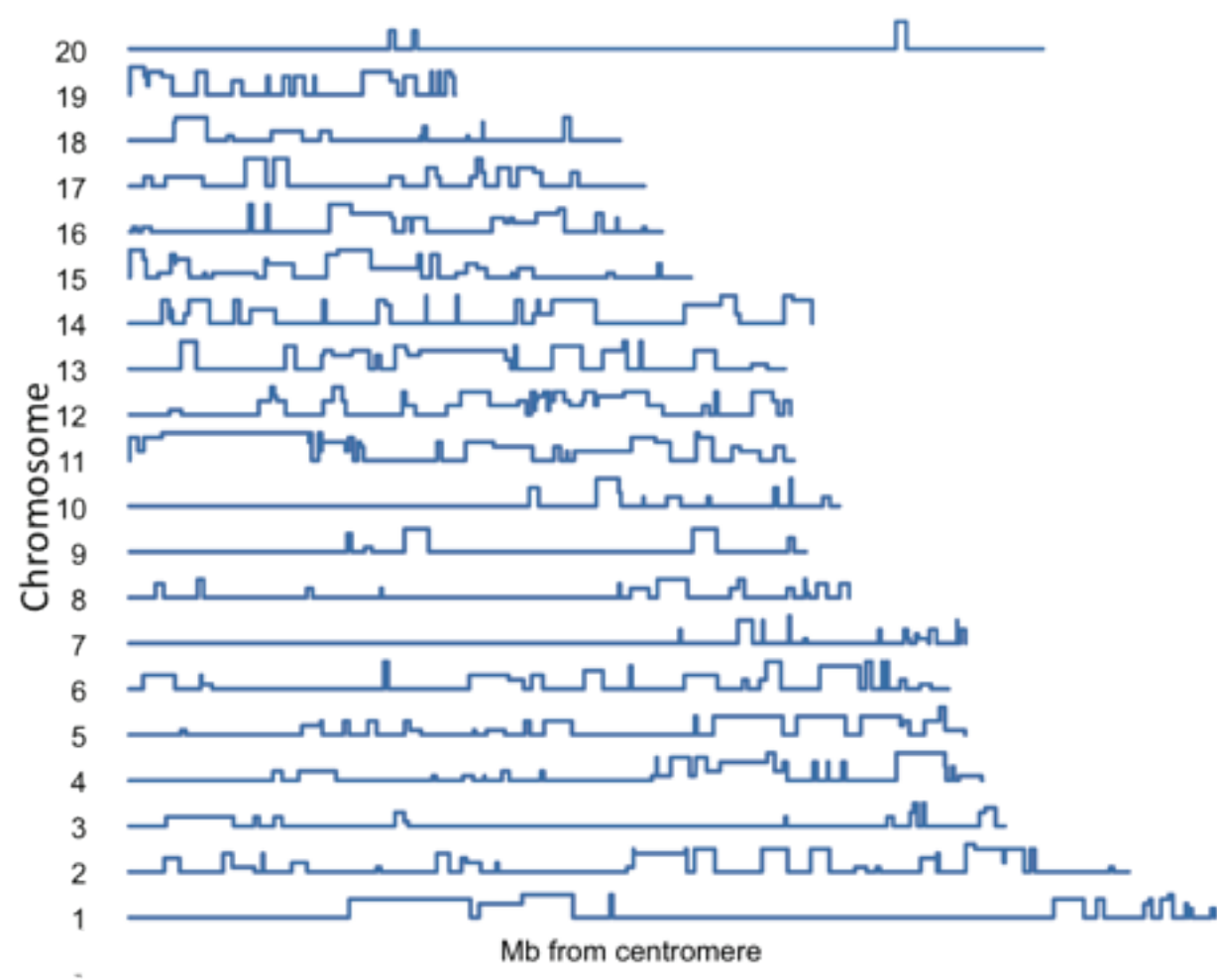

Supplement: Additional file 2 — Figure S1. Regional MAF analysis. Segregating regions were analyzed for MAF. Regions are classified with a MAF of 1 - 6 and are displayed as plateaus corresponding to the segregating region. [file 1471-2156-11-113-S2.PDF]
