# Supplementary material for: Genomic mapping of social behavior traits in a F2 cross derived from mice selectively bred for high aggression
Source: BMC Genet. 2010 Dec 31;11:113. doi: 10.1186/1471-2156-11-113 (PMC3022667; doi:10.1186/1471-2156-11-113)
Supplement: Additional file 3 — Table S2. List of 154 SNPs used in the final NC900 × B6 F2 map with known physical (Mb) and linkage (cM) positions from the Wellcome-CTC Mouse Strain SNP Genotype Set http://www.well.ox.ac.uk/mouse/INBREDS. [file 1471-2156-11-113-S3.DOC]

**Additional File 1, Table S2. List of 154 SNPs used in the final NC900 x B6 F2 map with known physical (Mb) and linkage (cM) positions from the Wellcome-CTC Mouse Strain SNP Genotype Set (**[**http://www.well.ox.ac.uk/mouse/INBREDS**](http://www.well.ox.ac.uk/mouse/INBREDS)**).**

| **SNP name** | **Chr** | **Mb location** | **cM location** |  | **SNP name** | **Chr** | **Mb location** | **cM location** |
| --- | --- | --- | --- | --- | --- | --- | --- | --- |
| rs13475701 | 1 | 4.49 | 0.10 |  | rs6256504 | 5 | 38.52 | 24.71 |
| gnf01.033.663 | 1 | 37.17 | 16.62 |  | rs3707918 | 5 | 72.13 | 43.34 |
| rs13472794 | 1 | 67.52 | 36.05 |  | rs13460234 | 5 | 89.85 | 57.53 |
| rs3670389 | 1 | 88.56 | 51.99 |  | rs3706737 | 5 | 96.53 | 64.85 |
| rs3667720 | 1 | 120.68 | 61.06 |  | rs13478466 | 5 | 111.80 | 73.63 |
| rs3703729 | 1 | 134.38 | 71.66 |  | rs3721911 | 5 | 138.88 | 96.54 |
| gnf01.151.323 | 1 | 151.79 | 78.27 |  | rs6284348 | 5 | 142.68 | 101.59 |
| rs13476229 | 1 | 169.24 | 93.68 |  | rs13478602 | 6 | 3.80 | 0.00 |
| rs3658234 | 1 | 175.00 | 99.30 |  | petM-02094-1 | 6 | 17.71 | 7.06 |
| rs3681873 | 1 | 183.94 | 107.47 |  | rs3678887 | 6 | 32.39 | 13.01 |
| rs3666905 | 1 | 187.55 | 112.13 |  | rs13478727 | 6 | 43.99 | 23.58 |
| rs13476312 | 1 | 193.71 | 121.48 |  | rs13478839 | 6 | 78.16 | 39.72 |
| rs13476334 | 2 | 7.29 | 3.18 |  | rs4226048 | 6 | 84.11 | 45.90 |
| rs13476348 | 2 | 11.09 | 8.96 |  | rs6226609 | 6 | 95.15 | 52.12 |
| gnf02.013.589 | 2 | 16.61 | 14.07 |  | rs13478934 | 6 | 102.55 | 59.70 |
| rs13476409 | 2 | 30.62 | 25.98 |  | mCV24115224 | 6 | 115.08 | 66.69 |
| rs6268714 | 2 | 57.64 | 35.46 |  | UT_6_123.37228 | 6 | 122.11 | 72.19 |
| rs6345656 | 2 | 74.67 | 48.89 |  | rs3688358 | 6 | 132.43 | 79.98 |
| rs13476654 | 2 | 97.29 | 60.30 |  | rs3725987 | 6 | 138.35 | 84.12 |
| rs3144393 | 2 | 118.27 | 70.43 |  | rs13479099 | 6 | 148.13 | 93.88 |
| rs13476878 | 2 | 160.23 | 91.03 |  | mCV22975338 | 7 | 22.38 | 11.75 |
| rs3143843 | 2 | 169.46 | 98.04 |  | rs6217275 | 7 | 31.59 | 18.77 |
| rs3663409 | 3 | 31.99 | 15.11 |  | rs3719256 | 7 | 38.61 | 23.53 |
| rs13477174 | 3 | 68.24 | 34.08 |  | rs3717846 | 7 | 58.40 | 30.34 |
| rs3670634 | 3 | 86.94 | 42.72 |  | rs3676254 | 7 | 66.57 | 38.01 |
| rs3663873 | 3 | 109.99 | 56.77 |  | rs3656205 | 7 | 75.34 | 45.44 |
| rs6212614 | 3 | 120.24 | 62.45 |  | rs13479422 | 7 | 91.89 | 53.39 |
| rs13477430 | 3 | 134.90 | 70.82 |  | rs13479471 | 7 | 108.99 | 65.05 |
| rs13477462 | 3 | 142.53 | 76.82 |  | rs13479520 | 7 | 122.59 | 77.99 |
| CEL-3_159340478 | 3 | 157.72 | 93.16 |  | rs13479540 | 7 | 128.36 | 85.72 |
| rs3674982 | 4 | 6.82 | 1.28 |  | rs6216320 | 7 | 139.60 | 94.26 |
| rs13477622 | 4 | 28.51 | 16.51 |  | CEL-8_7689226 | 8 | 7.06 | 0.91 |
| rs6232550 | 4 | 41.16 | 26.92 |  | rs13479657 | 8 | 24.62 | 14.69 |
| rs3725792 | 4 | 44.15 | 30.38 |  | rs13479731 | 8 | 42.07 | 23.35 |
| rs3715031 | 4 | 54.64 | 37.50 |  | rs13479844 | 8 | 75.47 | 38.39 |
| rs13477745 | 4 | 64.48 | 46.23 |  | rs13480010 | 8 | 116.45 | 69.26 |
| mCV23905937 | 4 | 98.48 | 64.56 |  | rs13480071 | 9 | 12.39 | 0.37 |
| rs6324470 | 4 | 104.94 | 69.21 |  | rs13480109 | 9 | 25.78 | 8.21 |
| rs3675629 | 4 | 120.08 | 77.90 |  | rs13480128 | 9 | 32.67 | 16.47 |
| rs4224808 | 4 | 131.96 | 86.58 |  | rs8259427 | 9 | 45.00 | 29.95 |
| CEL-4_149694865 | 4 | 150.30 | 101.35 |  | rs6239320 | 9 | 71.69 | 44.80 |
| CEL-5_5867251 | 5 | 5.87 | 2.21 |  | rs6213724 | 9 | 79.80 | 52.13 |
| rs3693453 | 5 | 13.62 | 5.05 |  | rs13480408 | 9 | 108.84 | 73.96 |
|  | | | |  |  |  |  |  |
| **Additional File 1, Table S2 (continued)** | | | |  |  |  |  |  |
| **SNP name** | **Chr** | **Mb location** | **cM location** |  | **SNP name** | **Chr** | **Mb location** | **cM location** |
| rs13480429 | 9 | 113.64 | 81.46 |  | rs3692586 | 15 | 42.53 | 15.31 |
| rs6316481 | 9 | 118.97 | 89.24 |  | CEL-15_58115663 | 15 | 57.93 | 24.93 |
| rs13480578 | 10 | 34.64 | 17.16 |  | rs13482636 | 15 | 69.66 | 33.61 |
| CEL-10_58149652 | 10 | 58.36 | 27.70 |  | rs3710055 | 15 | 88.01 | 46.08 |
| rs3717445 | 10 | 82.88 | 46.58 |  | rs6326790 | 15 | 90.60 | 52.10 |
| rs13480707 | 10 | 92.30 | 54.01 |  | rs3090912 | 16 | 5.85 | 0.10 |
| rs13480754 | 10 | 107.37 | 62.80 |  | rs4178513 | 16 | 40.52 | 32.74 |
| rs6199956 | 11 | 50.50 | 30.08 |  | rs6324074 | 16 | 58.68 | 39.15 |
| rs13481054 | 11 | 59.88 | 36.19 |  | rs3710273 | 16 | 71.69 | 47.29 |
| mCV22728592 | 11 | 75.32 | 47.30 |  | rs4212159 | 16 | 84.26 | 54.41 |
| rs8270290 | 11 | 97.03 | 64.64 |  | rs13482920 | 17 | 21.67 | 15.88 |
| rs3705163 | 11 | 106.71 | 81.97 |  | rs3023442 | 17 | 31.90 | 22.29 |
| rs6407460 | 11 | 113.21 | 90.80 |  | rs6395919 | 17 | 48.83 | 35.59 |
| CEL-11_118234030 | 11 | 118.38 | 96.66 |  | rs3663966 | 17 | 76.52 | 57.94 |
| rs6225272 | 12 | 27.46 | 13.90 |  | rs13483140 | 17 | 82.89 | 64.41 |
| rs13481491 | 12 | 58.58 | 30.11 |  | rs3696168 | 17 | 88.28 | 69.87 |
| rs3709008 | 12 | 71.54 | 36.75 |  | rs13483233 | 18 | 18.33 | 7.97 |
| rs3725854 | 12 | 78.00 | 43.15 |  | rs6313313 | 18 | 41.88 | 23.17 |
| rs13481604 | 12 | 93.51 | 56.41 |  | rs3722312 | 18 | 52.56 | 30.13 |
| rs6390948 | 12 | 104.15 | 66.83 |  | rs4211918 | 18 | 64.18 | 44.85 |
| rs13459139 | 13 | 12.10 | 1.87 |  | rs13483438 | 18 | 74.45 | 59.02 |
| rs13481734 | 13 | 26.52 | 9.63 |  | rs13483542 | 19 | 15.30 | 7.76 |
| rs13481780 | 13 | 40.76 | 17.40 |  | rs6237466 | 19 | 31.69 | 29.11 |
| rs4229749 | 13 | 51.89 | 24.30 |  | rs3703185 | 19 | 38.31 | 36.18 |
| rs3678784 | 13 | 61.65 | 32.38 |  | rs8257588 | 19 | 47.41 | 42.99 |
| mCV24625340 | 13 | 80.80 | 45.64 |  | mCV24736382 | 19 | 54.41 | 51.49 |
| CEL-13_105894110 | 13 | 105.35 | 61.86 |  | rs3718998 | 19 | 60.09 | 57.86 |
| rs13482019 | 13 | 109.16 | 67.16 |  | rs13483724 | X | 30.63 | 14.9137 |
| rs13482096 | 14 | 21.09 | 7.50 |  | rs13483748 | X | 45.25 | 22.853 |
| rs8251329 | 14 | 49.48 | 19.25 |  | rs13483770 | X | 53.99 | 29.028 |
| rs3712401 | 14 | 61.78 | 26.52 |  | CEL-X_91222960 | X | 94.42 | 43.283 |
| rs3709178 | 14 | 79.10 | 39.33 |  | rs13483992 | X | 124.11 | 55.715 |
| rs6179144 | 14 | 95.77 | 45.55 |  | CEL-X_133525088 | X | 137.07 | 73.1097 |
| rs13482404 | 14 | 116.10 | 60.78 |  | CEL-X_154259201 | X | 157.89 | 87.456 |
| rs13459145 | 15 | 6.96 | 1.34 |  |  | | | |
